# COVID-19 Vaccination and Disability in Ethiopia: Ensuring No One is Left Behind

**DOI:** 10.4314/ejhs.v32i3.25

**Published:** 2022-05

**Authors:** Abubeker Alebachew Seid

**Affiliations:** 1 Department of Nursing, College of Medicine and Health Science, Samara University, Samara, Ethiopia

***TO THE EDITOR:*** Globally, coronavirus disease 2019 (COVID-19) pandemic with re-occurring waves and new variants, and the subsequent health care crisis continues despite the unprecedented scientific achievement on vaccine development. As of January 27, 2022, the number of globally recorded COVID-19 cases and death is over 360 million and 5.6 million respectively (1). Ethiopia records 463,465 cases and 7,292 deaths at the same time (2).

Data from WHO and other sources showed that there are nearly 1 billion people with disabilities globally (3). This prevalence of disability is higher in lower income countries than in higher income countries. In Ethiopia, official data showed that about 15% (more than 15 million) of the population have a certain kind of disability that requires attention in every step of their daily life. People with disabilities might be at greater risk of contracting COVID-19 and poorer outcomes than the general population due to different contributing factors. Barriers to implementing basic hygiene measures, difficulty in enacting social distancing, barriers to accessing public health information and health care marginalization, and high prevalence of chronic diseases are the major factors responsible for high risk and poor COVID-19 outcomes in people with disabilities (4)(5). Consequently, those groups of people require greater attention in every step of measures taken to control the pandemic.

COVID-19 vaccines play an important role in reducing the number of cases, thereby reducing mortality and related health care costs. COVID-19 emergency vaccines approved by the World Health Organization (WHO) started to utilize at the end of 2020 and so far, (as of January 27, 2022) 60.7% of the world population and 9,372.021 people in Ethiopia has received at least one dose of COVID-19 vaccine. Unluckily, reports showed that the proportion of people vaccinated in the least developing countries (such as Africa) is very low (less than 10%), while it reaches 66.76% in South America, 62.94% in Europe, 60.32% in Asia, and 59.96% in North America (6). While the world is in a race of vaccination and reducing the impact of the pandemic, little or no attention has been given to people with disabilities, especially in low-income countries like Ethiopia. In Ethiopia, the National COVID-19 Vaccine Deployment Plan did not clearly consider people with disabilities in the national prioritization of the target population. In addition, there is no available evidence on the status of vaccination in people with disabilities, even no readiness is presumed to break this gap in Ethiopia.

To inform government agencies and policymakers on disability-friendly consideration in the vaccination program, to inform non-governmental and other concerned bodies/organizations working on disability to premier the agenda, and to inform people with disabilities to be actively involved and get vaccinated and not left behind, there is a need for continuous alarming of all bodies.

People with disabilities need to be considered in the prevention and treatment of, and preparedness for, current and future pandemic control measures. Health care planners and decision-makers have to take considerations always in all steps of COVID-19 control measures and disability-friendly service provision actions. The government, along with profit or non-profit private sectors, should consider initiating such inclusive approaches while planning and implementing health care services like COVID-19 vaccination. There should be efforts to expand the collection, analysis, and regular update of data on people with disabilities. Stakeholders should also encourage and involve in the “always consideration” of people with disabilities and ensure that services provided are accessible to everyone. In the digitalization of essential services like health care, these groups of peoples have to be considered as well as information communication gaps need to be addressed appropriately.

Last but not least, people with disabilities living in high-risk situations such as homeless, those in prison, and very remote areas should also be given increased attention to ensure no one is left invisible and behind.
